# Long-term associations between perinatal factors and white matter microstructure at 8–10 years

**DOI:** 10.3389/fnhum.2026.1729276

**Published:** 2026-02-13

**Authors:** Injoong Kim, Omar Azrak, Mark Foster, Emil Cornea, Sang Kyoon Park, Yoonmi Hong, Martin Styner, John H. Gilmore

**Affiliations:** 1 Department of Radiology, Veterans Health Service Medical Center, Seoul, Republic of Korea; 2 Department of Psychiatry, University of North Carolina at Chapel Hill, Chapel Hill, NC, United States; 3 Department of Biomedical Engineering, University of Basel, Basel, Switzerland

**Keywords:** brain development, diffusion tensor imaging, perinatal factors, school aged children, white matter microstructural changes

## Abstract

**Background:**

While perinatal factors are known to influence brain development, their long-term impact on white matter microstructure remains incompletely understood. Previous studies using tract-based spatial statistics (TBSS) have shown limited associations between neonatal measures and later white matter development.

**Methods:**

We investigated associations between perinatal factors (birth weight [BW], gestational age [GA], and head circumference at birth [HC]) and white matter microstructure in 117 children aged 8–10 years from the UNC Early Brain Development Study cohort. Diffusion tensor imaging (DTI) data were analyzed using a fiber tract-based framework examining 54 major white matter tracts. Statistical analyses were performed using a functional analysis of fiber tract profiles.

**Results:**

GA and BW showed widespread patterns of significant associations with white matter microstructure (38 and 36 out of 54 tracts, respectively), whereas HC showed limited associations (3 out of 54 tracts). *Post hoc* univariate analyses revealed stronger associations with axial diffusivity (AD) compared to radial diffusivity (RD) or fractional anisotropy (FA). AD associations with BW, GA, and HC were observed in 30, 31, and 8 tracts, respectively.

**Conclusion:**

Using a fiber tract-based analysis approach, we found that GA and BW were associated with widespread patterns of differences in white matter organization at school age, whereas HC showed limited associations. Associations involving AD were most consistently observed across tracts, suggesting that these perinatal factors may be related to variation in axonal characteristics of white matter. Overall, our findings indicate that early-life biological measures are related to later white matter microstructure, although further work is needed to clarify the developmental mechanisms underlying these associations.

## Introduction

Pre- and postnatal brain development is a complex process that is under active investigation, and it is a continuous and dynamic process that extends well into early adulthood (Stiles, 2008). Advances in magnetic resonance imaging (MRI) have provided researchers with tools to investigate developmental changes of cerebral gray and white matter. Diffusion tensor imaging (DTI) has been shown to capture white matter development, which is intrinsically linked to changes in the brain’s efficiency and related cognitive, behavioral, and psychopathological outcomes (Mabbott et al., 2006; Nagy et al., 2004; Filley and Fields, 2016). DTI characterizes white matter microstructure by measuring the orientation and integrity of white matter tracts (Mukherjee et al., 2008). This technique enables *in vivo* examination of tissue microstructure and provides quantifiable metrics relevant to both brain development and injury (Pecheva et al., 2018). Metrics obtained from DTI are highly sensitive to developmental shifts and white matter changes associated with prematurity and perinatal risk factors (Anjari et al., 2009; Ball et al., 2010; Chau et al., 2009; Zwicker et al., 2013). White matter pathways play a central role in the emergence of cognitive and behavioral functions, and their maturation follows a protracted, region-specific developmental course (Imperati et al., 2011; Lebel and Deoni, 2018). Building upon this, Nivins et al. (2023) studied how neonatal non-imaging measures (birth weight [BW], gestational age [GA], and head circumference at birth [HC]) were associated with imaging-derived measures of brain volume and white matter microstructure at school age (9–10 years). Notably, only birth weight showed some predictive value for white matter microstructure via the tract-based spatial statistics (TBSS) analysis in that study. In contrast, BW, GA, and HC are well-established specific indicators of fetal growth and early brain development, and prior studies have shown their associations with later white matter organization and neurodevelopmental outcomes (Jin et al., 2019; Solis-Urra et al., 2022). Furthermore, studying children aged 8–10 years is particularly informative, as this developmental period is characterized by relatively stable white matter maturation prior to pubertal changes, thereby reducing additional developmental variability (Lebel and Deoni, 2018; Simmonds et al., 2014). Methodologically, regional or white matter skeleton-based approaches often lack the sensitivity and spatial precision needed to detect tract-specific microstructural alterations, whereas fiber-tract–based analyses provide greater anatomical specificity and improved sensitivity (Short et al., 2022). Moreover, whereas prior studies such as Nivins et al. (2023) primarily focused on term-born children, our inclusion of participants spanning the full gestational age spectrum enables a more comprehensive evaluation of perinatal influences across a broader developmental range. Motivated by these limitations, the goal of the current study was to extend previous findings by applying a more spatially sensitive fiber tract-based DTI analysis framework (Verde et al., 2014) in a cohort with broader variability in perinatal factors, allowing comparison with results reported by Nivins et al. (2023).

Here, we aimed to investigate how common neonatal indicators of health (BW, GA, and HC) and brain development are related to DTI-based measurements of white matter microstructure in children aged 8–10 years.

## Materials and methods

### Participants

The UNC Early Brain Development Study (EBDS) database is a longitudinal dataset that has tracked children from prenatal stages, combining imaging data with cognitive and behavioral assessments throughout the course of their postnatal brain development (Knickmeyer et al., 2008; Stephens et al., 2020). Parents were recruited from UNC Hospitals and Duke University Medical Center during the second trimester of pregnancy, when they provided written informed consent. Mothers were excluded from the study for major illness or use of illegal drugs during pregnancy. From the EBDS cohort, we retrospectively selected the participants with diffusion MRI scans collected at ages 8 to 10 years on a 3 T Siemens Tim Trio using either a three-shell diffusion MRI sequence (diffusion shell *b*-value [number of diffusion weighted images per shell]: 0 [13], 300 [8], 700 [32], 2000 [64]; 2 × 2 × 2 mm^3^ resolution) or a single-shell sequence (*b* = 0 [7], 1,000 [42]). The full MRI session lasted approximately 35–40 min, including a 7–10 min DTI acquisition depending on scan type (single-shell or multi-shell). In addition, perinatal clinical information (HC, BW, and GA) was obtained for all participants. A total of 148 participants were selected based on the above criteria. All study protocols were approved by the Institutional Review Board of the UNC at Chapel Hill, and written informed consent was obtained from the parents of all participants.

### MRI processing and analysis

The diffusion MRI processing and analysis were performed using an updated version of the UNC NA-MIC DTI tract analysis framework (Verde et al., 2014). A study-specific quality control protocol was applied to three-shell raw DTI data using the dmriprep (Dubos et al., 2023) module of the DMRIPlayground toolkit.^1^ For the single-shell raw DTI data, a similar protocol adapted for this single-shell 42-direction DTI data was applied using DTIPrep v1.2.9 (www.nitrc.org/projects/dtiprep). The protocols used in both dmriprep and DTIPrep included correction for motion, eddy-current and susceptibility artifacts, as well as rejection of DWI volumes exhibiting significant residual slice-wise or gradient-wise artifacts. As part of the quality control procedure, all scans were visually inspected for structural abnormalities, including prematurity-related lesions (e.g., periventricular leukomalacia, intraventricular hemorrhage, or major white matter injury). Participants with any such abnormalities were excluded, and only scans with normal-appearing brain anatomy were retained for analysis. After conducting the quality control protocol, 31 participants were excluded due to insufficient image quality and limited brain coverage. Brain masks were estimated from the average *b* = 0 image and manually edited. Manual editing was restricted solely to the brain mask refinement step following initial quality control procedures. Diffusion tensors were estimated using a weighted least-squares approach. The three-shell DTI datasets were registered first to the EBDS 4-6-year DTI atlas, which was subsequently registered to the EBDS 1 -2-year DTI atlas (Short et al., 2022). Fifty-four major white matter tracts were resampled at evenly spaced locations along each tract (1 mm tract sampling) in the 1-2-year DTI atlas space. These tracts were then propagated into each participant’s native space via deformable mapping by combining the deformation fields from the registration of the DTI datasets to the 4-6-year DTI atlas and from the registration of the 4-6-year DTI atlas to the 1-2-year DTI atlas. The single-shell DTI datasets were registered directly to the 1-2-year DTI atlas, and the same 54 tracts were propagated into each participant’s native space via deformable mapping using the deformation fields from these registrations. Diffusion metrics—fractional anisotropy (FA), axial diffusivity (AD), and radial diffusivity (RD)—were extracted at each fiber location resulting in fiber tract profiles. As an additional quality control step, participants were excluded from further association analyses for a given tract if their FA profile was only weakly correlated with the population tract average profile (correlation <0.7). A low correlation typically flags poor alignment of the subject’s DTI to the atlas for the respective fiber tract.

**Figure 1:**
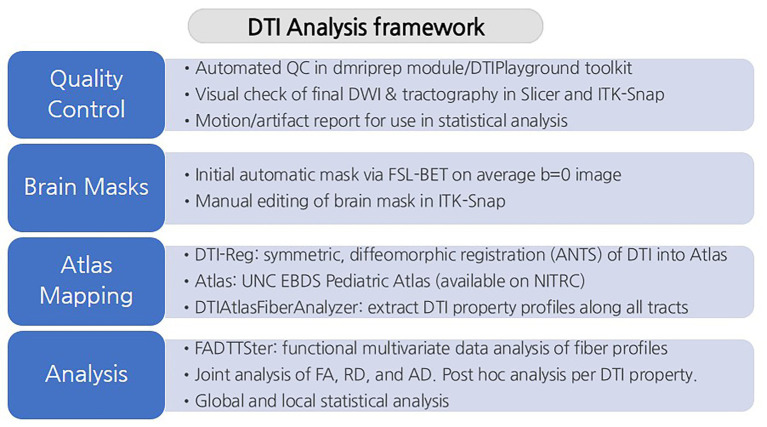
A schematic overview of the DTI analysis framework. The figure outlines the framework from quality control steps to statistical analysis of white matter tracts.

Statistical analysis of the fiber tract profiles was performed using FADTTS (Zhu et al., 2011), with sex and scan type (single-shell vs. three-shell acquisition) included as covariates, and global profile *p*-values were obtained for each tract. For each tract, we first conducted an omnibus multivariate analysis by including all three DTI metrics in a single model, followed by *post hoc* univariate analyses for FA, RD, and AD separately. Because our analyses were investigative, *p*-values were reported without correction for multiple comparisons across the fifty-four tracts. Strict multiple-comparison correction methods (such as Bonferroni) were not applied, and the findings are interpreted with appropriate caution given the potential for false-positive results. A schematic overview of the full processing and analysis pipeline is shown in Figure 1, providing a step-by-step visualization of the workflow described above.

## Results

Of the 148 participants, 31 were excluded during the quality control process, leaving 117 participants for analysis. Their baseline demographic and perinatal characteristics are summarized in Table 1. There were 94 participants scanned with the three-shell diffusion MRI sequence and 23 participants scanned with the single-shell diffusion MRI sequence. Gestational age was categorized following WHO guidelines as full term (37 ≤ GA < 42 weeks), moderate to late preterm (32 ≤ GA < 37 weeks), very preterm (28 ≤ GA < 32 weeks), and extremely preterm (GA < 28 weeks).

**Table 1:** Demographic characteristics of the children included in the DTI analysis.

Characteristic	N
Child sex	
Male	57 (48.7%)
Female	60 (51.3%)
Race	
White	93
Black or African American	24
Twin status	
Non-twin	52
Twin	65
Monozygotic	38
Dizygotic	27
Gestational age at birth	
Full term (37 ≤ GA < 42 weeks)	55
Moderate to late preterm (32 ≤ GA < 37 weeks)	53
Very preterm (28 ≤ GA < 32 weeks)	7
Extremely preterm (GA < 28 weeks)	2

Characteristic	Mean (standard deviation)	Range
Gestational age at birth (weeks)	36.46 (3.06)	27.42–41.14
Birth weight (g)	2740.64 (782.03)	790–4,732
Age at MRI scan (years)	9.24 (1.02)	8.0–10.9
Head circumference at birth (cm)*	32.54 (2.33)	24.0–37.75

*Data for head circumference were available for 113 participants (4 missing).

### Multivariate fiber-tract based analysis

**Figure 2:**
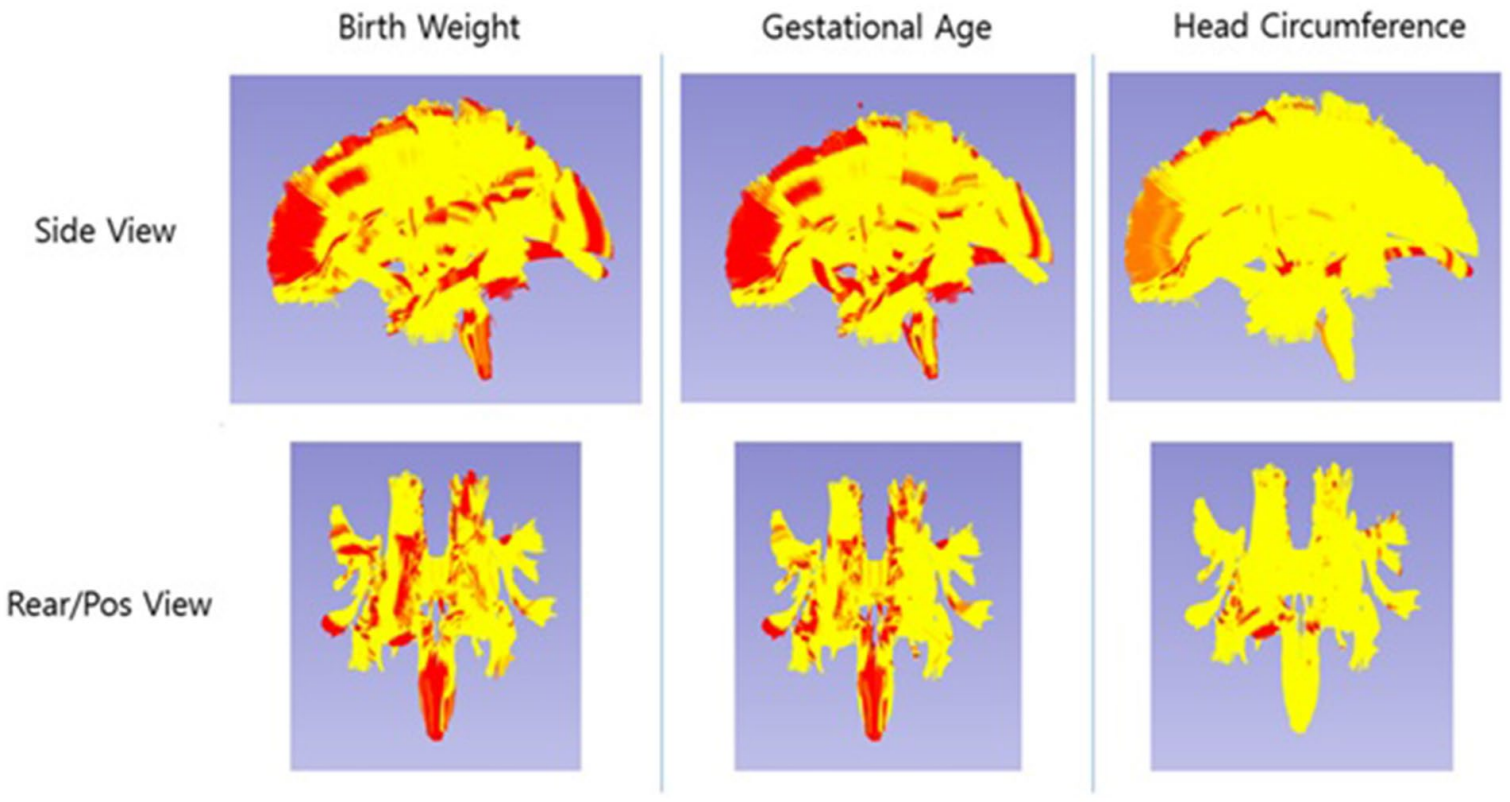
Spatial visualization of significant regions across the 54 white matter tracts. The yellow overlay represents the union of all tracts included in the analysis (white matter analysis mask). Red/orange regions indicate significant associations of tensor measures (FA, AD, and RD jointly) with perinatal factors (head circumference at birth, gestational age, and birth weight) (*p* < 0.05).

Among the three perinatal factors (BW, GA, and HC), GA exhibited the most extensive pattern of significant associations with white matter diffusion metrics, with significant global associations observed in 38 of the 54 tracts analyzed (*p* < 0.05; see Figure 2 and Table 2). BW (36 out of 54) showed a slightly lower number of globally associated tracts but also showed widespread significant associations. HC was associated with only 3 of the 54 tracts, indicating that the relationship between HC and white matter microstructure was limited.

**Table 2:** Number of tracts showing significant associations (*p* < 0.05) with perinatal factors across major white matter pathway groups.

Tract group	Birth weight	Gestational age	Head circumference
Arcuate fasciculus	3/6	2/6	0/6
Cingulum	2/4	3/4	0/4
Corpus callosum	5/8	5/8	0/8
Corticofugal tracts	7/8	8/8	1/8
Corticoreticular tracts	2/2	2/2	0/2
Corticospinal tracts	2/2	1/2	0/2
Corticothalamic tracts	7/10	7/10	1/10
Fornix	2/2	2/2	0/2
Inferior frontooccipital fasciculus	2/2	2/2	0/2
Inferior longitudinal fasciculus	1/2	1/2	1/2
Optic radiation	0/2	1/2	0/2
Optic tract	0/2	1/2	0/2
Superior longitudinal fasciculus	1/2	1/2	0/2
Uncinate fasciculus	2/2	2/2	0/2
Total (54 tracts)	36/54	38/54	3/54

Values are shown as *n*/total tracts per group. This summary highlights how many tracts within each major white matter pathway were significantly associated with birth weight, gestational age, or head circumference at birth. Full tract-level results are provided in Supplementary Table S1.

When examining associations by major white matter pathway groups (Table 2), GA and BW showed consistent associations across projection fibers (e.g., corticofugal, corticothalamic tracts), commissural pathways (e.g., corpus callosum), and limbic tracts (e.g., cingulum, fornix). In contrast, HC demonstrated sparse associations limited to a few pathways. These patterns indicate that GA and BW were associated across large-scale projection and commissural systems that support sensorimotor and integrative functions.

Sex and scan type were also significantly associated with the diffusion metrics in some white matter tracts. In the omnibus models, sex was significantly associated with diffusion metrics in 14 tracts, and scan type in 15 tracts (*p* < 0.05), indicating that both covariates contributed measurable variance to white matter microstructure across the cohort.

### *Post hoc* univariate analysis

In the *post hoc* univariate analysis, AD showed substantially more significant associations with the three perinatal factors than did RD or FA. AD was significantly associated with BW, GA and HC in 30, 31, and 8 of 54 white matter tracts, respectively. In contrast, RD showed significant associations in 5, 13, and 3 tracts, and FA in 12, 18, and 2 tracts for BW, GA, and HC, respectively.

These effects were most frequently observed in projection pathways (e.g., corticospinal, corticothalamic, and related corticofugal tracts), with additional but fewer associations in commissural (corpus callosum) and association fibers, and the fewest in limbic tracts (cingulum, fornix).

**Figure 3:**
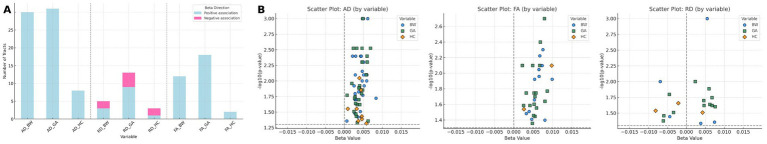
Associations between perinatal factors and white matter tract measures. **(A)** Directionality of beta coefficients for significant tracts associated with BW (birth weight), GA (gestational age), and HC (head circumference at birth), grouped by diffusion metrics (AD, RD, FA). Positive and negative associations are indicated in blue and pink, respectively. **(B)** Scatter plot visualization of significant tract-level associations. Each point represents a significant association, with color and shape indicating the variable (blue circle: BW, green square: GA, orange diamond: HC). The *x*-axis shows the beta coefficient for the association, indicating both the direction (positive or negative) and magnitude of the effect. The *y*-axis shows statistical significance expressed as –log_10_(*p*-value), where higher values indicate stronger evidence against the null hypothesis.

For tracts showing significant associations, FA and AD were consistently positively associated with BW, GA, and HC, indicating that larger perinatal measures were associated with higher FA and AD later. As shown in Figure 3, the majority of significant beta values for FA and AD fall within the positive range, whereas RD shows a more variable pattern. The magnitude of these beta coefficients was generally small across tracts.

## Discussion

In this study, we found evidence suggesting that neonatal health indicators, particularly gestational age (GA) and birth weight (BW), are associated with white matter microstructure at ages 8–10 years as measured by DTI. While previous studies have shown associations between perinatal factors and brain volumes in this age group (El Marroun et al., 2020; Davis et al., 2011; de Kieviet et al., 2012; Catena et al., 2019; Cheong et al., 2008), our findings extend this understanding to white matter organization. Notably, GA and BW showed a widespread pattern of positive associations with FA and AD, whereas HC demonstrated only limited relationships, suggesting that early growth measures may differentially influence later white matter development. These findings highlight the lasting impact of early-life biological status on white matter maturation and underscore the importance of considering perinatal factors when interpreting later neurodevelopmental outcomes.

Our findings both align with and differ from prior research in important ways. While earlier DTI studies using TBSS found limited associations between perinatal factors and diffusion metrics (Nivins et al., 2023; Ou et al., 2017), our fiber-tract analysis framework revealed broader association patterns. TBSS is well-known to serve as a hypothesis-generation tool due to its whole-brain, white matter skeleton-based analysis framework (Smith et al., 2006; Bach et al., 2014), but it is also known to be less sensitive than fiber-tract analysis frameworks such as ours or Automated Fiber Quantification (AFQ) (Bach et al., 2014; Yeatman et al., 2012; Kuchling et al., 2018; Wang et al., 2022). This may reflect the greater sensitivity of the fiber-tract-based analysis in detecting associations between perinatal factors and white matter microstructure, compared to previous TBSS-based studies (Nivins et al., 2023; Ou et al., 2017).

In contrast to prior studies showing strong associations between birth HC and later brain volume measures (Catena et al., 2019; Cheong et al., 2008), we found that HC was associated with only a small number of white matter tracts in our tract-based DTI analysis. This pattern suggests that HC may be more closely related to global brain size than to tract-specific microstructural organization, consistent with the view that volumetric and diffusion-based microstructural measures reflect distinct aspects of neurodevelopment.

Our sample includes a broader range of gestational ages, from extremely preterm to full term, which may help explain the widespread associations with GA observed in our results, in contrast to studies such as Nivins et al. (2023) that focused primarily on term-born children. Importantly, among the 62 preterm participants, the majority were moderate or late preterm (16 moderate and 37 late), with a small number of very (*n* = 7) or extremely preterm children (*n* = 2). This distribution indicates that our findings are unlikely to be driven by a highly premature subgroup. Including children born preterm allowed us to assess associations across a broader developmental spectrum and to capture white matter variability related to lower GA. This interpretation aligns with previous literature showing that preterm birth—particularly at lower gestational ages—is associated with reduced FA, elevated RD, and delayed microstructural maturation (Kelly et al., 2016; Thompson et al., 2014; Young et al., 2018). Accordingly, we focus on the broader pattern of associations across the white matter network rather than interpreting each tract individually. The widespread involvement of major projection and association pathways suggests that perinatal factors may exert diffuse influences on the development of long-range connectivity, consistent with the global nature of early growth-related risks.

In the *post hoc* analysis, AD showed the greatest number of significant tract-level associations with perinatal factors compared to FA and RD, although the magnitude of these effects was generally small. Although the most rapid phases of axonal growth occur earlier in development, AD remains sensitive to residual variation in axonal organization that persists into late childhood (Genc et al., 2023; Dimond et al., 2020). This continued sensitivity likely explains why neonatal factors showed stronger and more widespread associations with AD than with RD, which is more specifically influenced by myelination processes (Song et al., 2005). Taken together, these findings suggest that higher GA and BW–and, to a lesser extent, HC–are associated with more advanced white matter organization in school-age children.

Several limitations warrant consideration. First, our moderate sample size (*n* = 117) and use of a single 3 T MRI scanner may limit the generalizability of our findings. Second, although the inclusion of children ranging from extremely preterm to full term allowed us to examine perinatal influences across a broad developmental spectrum, this heterogeneity may also introduce confounding effects related to differing medical and developmental trajectories. Third, neurodevelopment is shaped by complex interactions among genetic, environmental, and postnatal influences, none of which could be fully captured in the present analysis. In addition, although we included twin status as a covariate, within-pair relatedness was not modeled, which may have introduced non-independence in the data. Future work should incorporate analytic approaches that account for familial clustering (e.g., mixed-effects or GEE models). Finally, we did not apply formal multiple-comparison correction across tracts. Although white matter pathways are highly correlated and our analyses were investigative in nature—conditions under which traditional correction procedures may be overly conservative—this remains a limitation, and the findings should be interpreted with appropriate caution. Accordingly, the present results should be viewed as hypothesis-generating rather than as definitive evidence of tract-specific effects.

Future directions include expanding the analysis to the full EBDS cohort (over 300 participants scanned at age 8–10), which will allow for validating the current findings in a larger, more diverse sample once ongoing data harmonization is complete. Because these scans were acquired across three different 3 T Siemens MRI scanners, additional harmonization will be required to ensure consistency across scanners. In addition, the study plans to implement a more detailed analysis of white matter microstructure using the Neurite Orientation Dispersion and Density Imaging (NODDI) model, which will allow for a more refined and biologically specific understanding of white matter development in childhood.

In conclusion, we found that BW and GA—more so than HC—were associated with widespread patterns of differences in white matter organization in school-age children. These associations were most consistent for AD, suggesting sensitivity to long-term variation in axonal properties. Overall, our findings suggest that early-life biological factors may leave a measurable imprint on later white matter development.

## Data Availability

The data analyzed in this study is subject to the following licenses/restrictions: The dataset used in this study contains sensitive MRI and clinical information from child participants and is therefore subject to privacy and ethical restrictions. Data sharing is limited by the terms of the Institutional Review Board (IRB) approval from the University of North Carolina at Chapel Hill. Access to the dataset can be provided upon reasonable request to the UNC Early Brain Development Study (EBDS) research team and with appropriate IRB authorization. Requests to access these datasets should be directed to John H. Gilmore, john_gilmore@med.unc.edu.

## Footnotes

1
https://github.com/NIRALUser/DTIPlayground
