# Supplementary material for: Long-term associations between perinatal factors and white matter microstructure at 8–10 years
Source: Front Hum Neurosci. 2026 Feb 13;20:1729276. doi: 10.3389/fnhum.2026.1729276 (PMC12947122; doi:10.3389/fnhum.2026.1729276)
Supplement: Supplementary file 1 [file Table_1.DOCX]

Table S1. Fiber tract-based analysis results showing global p-values for all 54 white matter tracts. Light green shading indicates tracts with significant associations (p < 0.05) with birth weight, gestational age, or head circumference at birth. This table provides the full tract-level results complementing the summary data presented in Table 2.

| **White matter tract** | **Birth Weight (p)** | **Gestational Age (p)** | **Head Circumference (p)** |
| --- | --- | --- | --- |
| Arcuate Fasciculus Left Frontoparietal | 0.011 | 0.034 | 0.346 |
| Arcuate Fasciculus Left Frontotemporal | 0.049 | 0.069 | 0.327 |
| Arcuate Fasciculus Left Temporoparietal | 0.028 | 0.046 | 0.652 |
| Arcuate Fasciculus Right Frontoparietal | 0.178 | 0.522 | 0.351 |
| Arcuate Fasciculus Right Frontotemporal | 0.110 | 0.103 | 0.224 |
| Arcuate Fasciculus Right Temporoparietal | 0.397 | 0.336 | 0.357 |
| Cingulum (Cingulate) Left | 0.021 | 0.024 | 0.408 |
| Cingulum (Cingulate) Right | 0.022 | 0.032 | 0.521 |
| Cingulum (Hippocampal) Left | 0.114 | 0.042 | 0.099 |
| Cingulum (Hippocampal) Right | 0.232 | 0.119 | 0.417 |
| Corpus Callosum Body | 0.006 | 0.022 | 0.314 |
| Corpus Callosum Genu | 0.009 | 0.001 | 0.173 |
| Corpus Callosum Motor segment | 0.130 | 0.088 | 0.329 |
| Corpus Callosum Parietal segment | 0.018 | 0.027 | 0.255 |
| Corpus Callosum Premotor segment | 0.008 | 0.007 | 0.114 |
| Corpus Callosum Rostrum | 0.090 | 0.084 | 0.546 |
| Corpus Callosum Splenium | 0.022 | 0.069 | 0.279 |
| Corpus Callosum Tapetum | 0.051 | 0.013 | 0.280 |
| Corticofugal Tract Left Motor | 0.004 | 0.008 | 0.168 |
| Corticofugal Tract Left Parietal | 0.016 | 0.019 | 0.266 |
| Corticofugal Tract Left Prefrontal | 0.002 | 0.001 | 0.043 |
| Corticofugal Tract Left Premotor | 0.055 | 0.027 | 0.235 |
| Corticofugal Tract Right Motor | 0.004 | 0.005 | 0.062 |
| Corticofugal Tract Right Parietal | 0.004 | 0.001 | 0.201 |
| Corticofugal Tract Right Prefrontal | 0.003 | 0.002 | 0.121 |
| Corticofugal Tract Right Premotor | 0.016 | 0.007 | 0.162 |
| Corticoreticular Tract Left | 0.018 | 0.006 | 0.423 |
| Corticoreticular Tract Right | 0.014 | 0.006 | 0.145 |
| Corticospinal Tract Left | 0.042 | 0.069 | 0.171 |
| Corticospinal Tract Right | 0.009 | 0.012 | 0.132 |
| Corticothalamic Tract Left Motor | 0.052 | 0.050 | 0.201 |
| Corticothalamic Tract Left Parietal | 0.006 | 0.005 | 0.087 |
| Corticothalamic Tract Left Prefrontal | 0.158 | 0.067 | 0.481 |
| Corticothalamic Tract Left Premotor | 0.160 | 0.129 | 0.535 |
| Corticothalamic Tract Left Superior | 0.012 | 0.040 | 0.172 |
| Corticothalamic Tract Right Motor | 0.009 | 0.006 | 0.043 |
| Corticothalamic Tract Right Parietal | 0.005 | 0.002 | 0.051 |
| Corticothalamic Tract Right Prefrontal | <0.001 | <0.001 | 0.106 |
| Corticothalamic Tract Right Premotor | 0.018 | 0.029 | 0.195 |
| Corticothalamic Tract Right Superior | 0.008 | 0.007 | 0.092 |
| Fornix Left | 0.037 | 0.003 | 0.245 |
| Fornix Right | 0.015 | 0.007 | 0.383 |
| Inferior Frontoccipital Fasciculus Left | 0.008 | 0.003 | 0.207 |
| Inferior Frontoccipital Fasciculus Right | 0.014 | 0.017 | 0.313 |
| Inferior Longitudinal Fasciculus Left | 0.016 | 0.008 | 0.018 |
| Inferior Longitudinal Fasciculus Right | 0.109 | 0.100 | 0.559 |
| Optic Radiation Left | 0.332 | 0.149 | 0.704 |
| Optic Radiation Right | 0.209 | 0.047 | 0.323 |
| Optic Tract Left | 0.053 | 0.020 | 0.447 |
| Optic Tract Right | 0.521 | 0.254 | 0.705 |
| Superior Longitudinal Fasciculus II Left | 0.142 | 0.269 | 0.657 |
| Superior Longitudinal Fasciculus II Right | 0.016 | 0.015 | 0.242 |
| Uncinate Fasciculus Left | 0.019 | 0.006 | 0.303 |
| Uncinate Fasciculus Right | 0.036 | 0.018 | 0.530 |
